# ID 151 - Derivados da *Cannabis* e seus Análogos Sintéticos para Transtorno do Espectro Autista

**DOI:** 10.5327/2237-9622.2025.v34s1.151

**Published:** 2025-11-25

**Authors:** Roberta Borges Silva, Carolina de Oliveira Cruz Latorraca, Rafael Leite Pacheco, Ana Luiza Cabrera Martimbianco, Camila Monteiro Cruz, Cecília Menezes Farinasso, Isabela Porto de Toledo, Patrícia do Carmo Silva Parreira, Roberta Borges Silva, Verônica Colpani, Rachel Riera

**Keywords:** transtorno do espectro autista, TEA, *Cannabis*, canabidiol, revisão sistemática

## Abstract

**Introdução:**

A prevalência de transtorno do espectro autista (TEA) no Brasil e no mundo é estimada em 1% e dados do Sistema Único de Saúde (SUS) apontam que essas pessoas realizaram 9,6 milhões de atendimentos ambulatoriais em 2021. O uso terapêutico de derivados da e seus análogos sintéticos tem sido proposto para condições como demência, epilepsia, fibromialgia, HIV/aids, náusea e vômitos relacionados à quimioterapia, à esquizofrenia e à síndrome de Tourette. O objetivo deste estudo foi avaliar os efeitos dos derivados da e seus análogos sintéticos para pessoas com TEA.

**Método:**

Revisão sistemática, conduzida no Nats/NEv do Hospital Sírio-Libanês, seguindo as recomendações metodológicas do Cochrane Handbook for Systematic Reviews of Interventions e relatada de acordo com o Preferred Reporting Items for Systematic Reviews and Meta-Analyses (PRISMA 2020). O protocolo foi registrado prospectivamente na base PROSPERO (https://www.crd.york.ac.uk/prospero/display_record.php?RecordID=468300). Em 7/12/2023, foi realizada busca ampla e sensível em bases eletrônicas (ADOLEC, CENTRAL, Embase, Epistemonikos, LILACS, MEDLINE, PsycINFO), literatura cinzenta (DANS), registros de ensaios clínicos (clinicaltrials.gov e ICTRP-WHO) e busca manual em listas de referências. Foram considerados ensaios clínicos randomizados (ECR) que avaliaram derivados da e seus análogos sintéticos para pessoas com TEA comparado com nenhuma intervenção, placebo, lista de espera ou outras terapias ativas. Desfechos primários: avaliação global, gravidade dos sintomas eventos adversos graves; secundários: qualidade de vida, comportamento adaptativo e eventos adversos. O risco de viés dos ECR foi avaliado com Cochrane RoB 2.0 e RoB-ME e a certeza da evidência foi avaliada pela abordagem GRADE.

**Resultados:**

Foram identificadas 71.073 referências e, após o processo de seleção, 11 ECR (2 com resultados disponíveis e 9 em andamento), avaliando 214 participantes com idade entre 5 e 21 anos, foram incluídos. Quando comparado ao placebo, a solução oral de extrato integral de (solução oral, diluída em azeite de oliva na proporção CBD/THC = 2:1) pode aumentar a probabilidade de melhora no escore global dos sintomas em 12 semanas (RR 2,2; IC 95% 1,22 a 4,29; 1 ECR, 92 participantes; evidência de baixa certeza) e o risco de evento adversos é incerto (RR 1,98; IC 95% 0,18 a 21,45; 1 ECR, 92 participantes; evidência de certeza muito baixa). Quando comparada ao placebo, os efeitos da solução oral de extrato purificado de na avaliação global dos sintomas (RR 1,77; IC 95% 0,91 a 3,45; 1 ECR, 92 participantes; evidência de certeza muito baixa) e no risco de eventos adversos são incertos (RR 3,03; IC 95% 0,32 a 28,62; 1 ECR, 92 participantes; evidência de certeza muito baixa).

**Conclusão:**

De acordo com os resultados dos ECR disponíveis até o momento, as evidências sobre os benefícios e os riscos dos derivados da e seus análogos sintéticos para pessoas com TEA são limitadas (estudos pequenos e com baixa qualidade metodológica e para uma única apresentação de). Diante dessa limitação, é importante discutir a utilização desta intervenção no cenário nacional, considerando ainda que a apresentação de autorizada para comercialização no Brasil (como medicamento) não possui, em sua bula, indicação para TEA.

